# Evaluation of CAMPYLOBACTER QUIK CHEK™ rapid membrane enzyme immunoassay to detect *Campylobacter* spp. antigen in stool samples

**DOI:** 10.1186/s13099-021-00400-0

**Published:** 2021-01-22

**Authors:** Justine Franco, Lucie Bénejat, Astrid Ducournau, Francis Mégraud, Philippe Lehours, Emilie Bessède

**Affiliations:** 1 Centre National de Référence des Campylobacters et Helicobacters, Bordeaux University Hospital, Bordeaux, France; 2 INSERM U1053 Bordeaux Research In Translational Oncology, Bordeaux, France

**Keywords:** Immunoenzymatic techniques, Campylobacter rapid detection, Children, Gastroenteritis, Composite reference test

## Abstract

*Campylobacter spp.* enteritis is the most frequent bacterial enteritis in both adults and children and is sometimes a source of severe complications. Its diagnosis by culture suffers from a lack of sensitivity and delays the result, preventing an early initiation of optimal antibiotic therapy in some cases. Our aim was to test a new rapid immuno-enzymatic method for *Campylobacter spp.* diagnosis in comparison to a composite reference standard (CRS). Stool samples from the French National Reference Center for Campylobacter and Helicobacter were tested with the CAMPYLOBACTER QUIK CHEK™ (Abbott). The CRS used to consider a case positive for *Campylobacter spp.* was a positive culture and, in case of a negative culture, a positive result obtained with both an ELISA and a molecular test. One hundred and eight stools were included: 53 were positive according to the CRS. If performed alone, culture would have missed 5 cases which the CAMPYLOBACTER QUIK CHEK™ detected. Finally, the CAMPYLOBACTER QUIK CHEK™ showed a sensitivity of 96.2% and a specificity of 94.5% and is relevant for clinical practice. Given the characteristics of the new method, it can be used as a screening method for *Campylobacter spp.* detection.

## Introduction

Campylobacters are the main cause of bacterial diarrhea and one of the most widespread infectious diseases worldwide over the last 100 years [1, 2]. The incidence of campylobacteriosis is increasing in both high- and low-income countries [1]. According to the national health agency in France, Santé Publique France, it is the first cause of hospitalization and the third cause of death secondary to a foodborne infection in France [3]. This infection, usually caused by *Campylobacter jejuni* or *Campylobacter coli,* can also lead to severe gastrointestinal and extraintestinal manifestations, infectious or post-infectious, like the Guillain Barré syndrome which is responsible for neurological sequelae in 15 to 22% of cases [1, 4, 5]. The clinical signs of this bacterial intestinal infection are non-specific. Antibiotic therapy for campylobacteriosis is most effective when started within the first 3 days after the occurrence of the symptoms, in order to shorten the duration of the disease. Antibiotic treatment also shortened the gut carriage of Campylobacters and is indicated to reduce transmission in day-care centers and children's institutions [6]. The rapid identification of these bacteria can also guide the choice of antibiotic therapy in order to limit the selection pressure and other consequences related to the prescription of broad-spectrum antibiotics. Stool culture is the reference test to detect Campylobacters but the result is usually obtained in a minimum of 48 h. Moreover, culture is demanding and its sensitivity is low, in the range of 60% and 76% according to previous studies [7, 8]. Nowadays, several culture-independent diagnostic tests (CIDT) are available, giving faster results than culture as well as a better sensitivity and a good specificity. Among them, molecular methods (real-time PCRs) and some enzyme-linked immunosorbent assays (ELISAs) require additional automation to be performed and are technically demanding [8–10]. Immunochromatographic tests are easier to use but their reported sensitivity in some studies is lower [11–13]. The CAMPYLOBACTER QUIK CHEK™ (Abbott, Chicago, IL, USA) is a new membrane enzyme immunoassay (EIA) based on the very quick and easy qualitative detection of a thermotolerant Campylobacter-specific antigen in human stool specimens. It provides a result in less than 30 min. The aim of this study was to evaluate its performance.

## Materials and methods

This retrospective study was conducted in July 2019 at the French National Reference Center for Campylobacters and Helicobacters (NRCCH) located in the Bacteriology Laboratory at the Bordeaux University Hospital.

### Sample collection

One hundred and eight stools from the NRCCH collection were used in the study. Stools were collected previously from 84 patients at the Bordeaux University Hospital or from 24 ambulatory patients at a private laboratory (Exalab, Le Haillan, France). These 24 stools were transported at + 4 °C in a Cary-Blair medium. All stool cultures were requested by clinicians between 2016 and 2019 because of the patients’ symptomatology. Culture for Campylobacters was performed according to routine clinical procedures and then samples were all kept frozen at − 80 °C.

### CAMPYLOBACTER QUIK CHEK™

After thawing, all specimens were tested by the rapid membrane EIA, CAMPYLOBACTER QUIK CHEK™, following the manufacturers’ instructions. Briefly, this EIA consists of a cassette containing a membrane on which there is a control and a test strip. Specific antibodies against Campylobacter-specific antigen are present in the test strip, and anti-gamma immunoglobulin antibodies in the control strip. The amount of stool required to perform the test is 25 µL which is mixed in a dilution tube with 750 µL of diluent and 50 µL of conjugate. In this study, an antibody against Campylobacter-specific antigen coupled to horseradish peroxidase was used. After vortexing, 500 µL of the eluate was transferred to the sampling window of the cassette and incubated at room temperature for 15 min. Three hundred µL of wash buffer followed by two drops of substrate were added to the reading window. Interpretation of the test was performed after 10 min of incubation at room temperature. The test was interpreted as positive if both the test line and the control line were present, negative if only the control line was present, or uninterpretable if the control line was absent. Results were read by the naked eye by two independent observers blinded to the results of the other tests.

### Culture

Culture was performed on a Campylosel agar plate (bioMérieux, Marcy l’Etoile, France). Plates were incubated for a maximum of 3 days in a microaerobic atmosphere at 35 ± 2 °C. Colonies resembling Campylobacters colonies were directly identified by matrix-assisted laser desorption ionization–time of flight mass spectrometry (Bruker Daltonics, Bremen, Germany) [14].

### Real-time PCR

DNA extractions were performed using an Arrow Stool DNA kit (DiaSorin, Cypress, CA); no extraction control was used. A real-time PCR specific for *C. jejuni* and *C. coli*, targeting the *gyr*A gene was performed, as previously described, on all stool-culture negative samples [15].

### ELISA

The ELISA test (RIDASCREEN Campylobacter, r-biopharm AG, Darmstadt, Germany) is based on the detection of an antigen of *C. jejuni* and *C. coli* in the stool sample. It was used following the manufacturer’s instruction.

### Statistical analysis and composite reference standard (CRS)

To overcome the lack of sensitivity of culture and to better assess the performance of CAMPYLOBACTER QUIK CHEK™, a CRS was used. A positive case corresponded to a positive culture and, in case of a negative culture result, by simultaneous positivity of real-time PCR and ELISA.

### Clinical information

In case of discrepancy between a negative culture and a positive CAMPYLOBACTER QUIK CHEK™, the following data were collected from the intranet medical records: fever, presence of bloody diarrhea, results of abdominal imaging and blood tests, hospitalization, antibiotic therapy and eventual differential diagnosis.

### Ethics

All diagnostic methods were performed routinely. All patients were investigated in a hospital or private setting, according to good clinical practices. No informed consent for using human stool samples was requested of the patients. Therefore, to ensure subject anonymity, all directly identifiable patient data were removed from the present study.

## Results

According to the CRS, among the 108 stools, 53 were positive and 55 were negative. There was 100% agreement between the 2 observers for the interpretation of the results of the CAMPYLOBACTER QUIK CHEK™.

CAMPYLOBACTER QUIK CHEK™ was positive for 54 samples. It successfully detected 51 out of the 53 (96.2%) positive samples with the CRS (Table 1) and 46 out of the 48 (96%) positive samples with culture. Of the 8 positive CAMPYLOBACTER QUIK CHEK™ with negative culture, 5 were true positives according to the CRS and 3 were false positives, 2 with a negative real-time PCR and a positive ELISA, and one with both a negative ELISA and a negative molecular method (Table 2).

**Table 1:** Comparison between detection of Campylobacter using the CAMPYLOBACTER QUIK CHEK™ and the composite reference standard

	CRS +	CRS−	Total
CAMPYLOBACTER QUIK CHEK™ +	51	3	54
CAMPYLOBACTER QUIK CHEK™−	2	52	54
Total	53	55	108

*CRS* composite reference standard

**Table 2:** Profile observed according to the positive or negative result for all diagnostic tests applied

CRS	CAMPYLOBACTER QUIK CHEK™	Culture	RT-PCR	ELISA	Total	
+	+	+	N^a^	N^a^		46
	+	−	+	+		5
	−	+	+	N^a^		2
−	−	−	−	N^b^		52
	+	−	−	+		2
	+	−	−	−		1

*CRS* composite reference standard, *ELISA* enzyme-linked immunosorbent assay, *RT-PCR* Real-time PCR

N^a^ Not done because culture was positive, N^b^ Not done because RT-PCR was negative

CAMPYLOBACTER QUIK CHEK™ was negative for 54 specimens. Stool cultures as well as the CRS were negative for 52 of them and positive for the 2 others. These 2 specimens were considered as false negatives (Table 2).

According to the CRS, the sensitivity of CAMPYLOBACTER QUIK CHEK™ was 96.2%, (95% CI [92.6– 99.8]) and its specificity was 94.5%, (95% CI [90.2–98.8]).

Among the 5 true positive results with a positive CAMPYLOBACTER QUIK CHEK™ and a negative culture, 4 patients had a fever and 2 of them had bloody diarrhea. The 4 patients for whom information was available had a C-reactive protein (CRP) level greater than 75 mg/L. Two patients needed imaging which revealed a colitis. Two were hospitalized for this episode. Four patients received probabilistic antibiotic therapy for their diarrhea: 2 were treated with 3rd generation cephalosporins (3GC), one with fluoroquinolone and one with azithromycin. No other diagnosis was made for these patients. The false positive result, with CAMPYLOBACTER QUIK CHEK™ as the only positive result was obtained with a stool sample from a patient for whom a diagnosis of *Chlamydia trachomatis* infection was made and who presented a diarrhea*.* For the 2 false positive results by CAMPYLOBACTER QUIK CHEK™ where there was also a positive ELISA, information was available for only one patient. The patient suffered from a non-bloody diarrhea with fever symptoms which resolved spontaneously without antibiotic treatment.

## Discussion

The aim of our study was to evaluate the performance of a new EIA test, the CAMPYLOBACTER QUIK CHEK™, on a stool collection. In order to overcome the lack of sensitivity of stool culture previously reported, a CRS was used, combining positive culture and, in the case of a negative culture, both a positive molecular test and a positive ELISA. We did not perform the molecular method when culture was positive because of the excellent specificity with this latter method when MALDI-TOF gives a score up to 2 for the Campylobacter identified [14]. The diagnostic accuracy of the real-time PCR and the ELISA was previously assessed by Asselineau et al. In their study, a latent class model confirmed that these CIDTs were valid to evaluate a test in the absence of a correct gold standard [16]. Furthermore, despite the fact that the real-time PCR and ELISA chosen are not perfect gold standards, they are based on two different principles and are known to be reliable and reproducible to detect *C. jejuni* and *C. coli* in stool samples [8, 17]. The diagnostic accuracy of the CRS is unknown, but using these two different accurate techniques increases the likelihood that the CAMPYLOBACTER QUIK CHEK™ result will be a true positive or a true negative. The same kind of CRS was previously used to estimate the performance of two other immunochromatographic tests [11] for *Campylobacter* spp. antigen detection. A limitation of our retrospective study is that it was performed using a stool collection with a positivity rate of 50% which does not correspond to the current prevalence observed in a routine laboratory in developed countries. Thus, accuracy of the CAMPYLOBACTER QUIK CHEK™ was determined only in terms of sensitivity and specificity but not of predictive values.

CAMPYLOBACTER QUIK CHEK™ detected more Campylobacters than culture but the difference was not statistically significant (Table 2). Furthermore, the use of CAMPYLOBACTER QUIK CHEK™ allows the initiation of an earliest appropriate treatment since it gives a result in less than 30 min offering the possibility to treat the patients earlier when needed. Moreover, this result is interesting regarding the underestimated burden of campylobacteriosis. It is also interesting to note that patients with a true positive CAMPYLOBACTER QUIK CHEK™, a negative culture and a positive CRS were not pauci-symptomatic or asymptomatic. Their symptoms were not specific to a Campylobacter infection but were strongly suggestive of a bacterial intestinal infection. For all of these patients, a complete stool culture was performed and results were all negative for *Yersinia* sp*, Shigella* sp*, Salmonella* sp and *Campylobacter* spp other than *C. jejuni* and *C. coli*. In addition, no other differential diagnosis was made. The detection of DNA or antigen does not necessarily prejudge the viability and infectivity of the organism found but these clinical data reinforce the probability that the intestinal infection is indeed related to a Campylobacter and not due to an overly sensitive test as can be the case with some molecular tests [18].

Our study confirmed the work performed by Schnee et al*.* who showed that CAMPYLOBACTER QUIK CHEK™ is relevant for clinical practice. They evaluated the performance of this test on diarrheal stools from children aged 0–24 months, living in Bangladesh. They compared the CAMPYLOBACTER QUIK CHEK™ to a quantitative in-house PCR and showed an excellent performance of this test with a sensitivity of 95.7% and a specificity of 97% when the cycle threshold (Ct) of the qPCR was low [19]. A previous study, also conducted on a population of children aged 0–2 years in low-resource countries, estimated that below this low Ct value the amount of Campylobacter in the feces is sufficient to be associated with the diarrheal episode [20].

Another interesting point concerning the elements in the medical records is the fact that 4 patients received probabilistic antibiotic therapy, half of them with C3G, antibiotics which are not effective against thermotolerant Campylobacters. Performing a CAMPYLOBACTER QUIK CHEK™ test upon receipt of the stool sample at the laboratory would have allowed the prescription of an adequate antibiotic therapy at the time of initial management.

The potential problem of the CAMPYLOBACTER QUIK CHEK™ is its spectrum including only *C. jejuni* and *C. coli* detection. Even if the greater majority of campylobacteriosis are due to these 2 species, other Campylobacters especially *Campylobacter fetus* and *Campylobacter lari* can cause gastroenteritis. In this study, we performed the EIA on strains of 4 different *Campylobacter* species or related organisms (*Campylobacter upsaliensis, C. fetus, C. lari and Arcobacter butzleri*) and a cross-reactivity of the EIA was observed with *C. upsaliensis* and *C. lari* (data not shown) which are the 3rd and 4th most commonly isolated *Campylobacter* species in foodborne illnesses in the United States of America [21]. Indeed, Abbott in its latest intended added the detection of *C. lari* and *C. upsaliensis* in the instructions for use. However, this EIA does not detect *C. fetus* which is often responsible for more serious illnesses than those caused by *C. jejuni* and *C. coli* and causes bloodstream infections more commonly than gastroenteritis, even in patients at extreme ages [21]. It is important to note that, when this EIA is negative, the possibility of a campylobacteriosis cannot be ruled out, proving that culture on stools still needs to be performed to maximize the chance to detect all *Campylobacter* spp*.*

For the two false negative CAMPYLOBACTER QUIK CHEK™ cases, culture was positive and a *C. jejuni* and *C. coli* were isolated. These two results could be explained by the use of samples stored in a transport medium which is not recommended by the manufacturer. For two false positive results obtained with CAMPYLOBACTER QUIK CHEK™, culture and real-time PCR were negative but ELISA was positive. Following our CRS definition, these 2 stools were negative, but it is interesting to note that another molecular test (RIDA@GENE Bacterial Stool Panel, r-biopharm, Darmstadt, Germany) was performed on these 2 samples and was also positive for *C. jejuni.* This means that the specificity of the CAMPYLOBACTER QUIK CHEK™ was probably underestimated.

## Conclusion

CAMPYLOBACTER QUIK CHEK™ is a reliable test with a good performance to detect *C. jejuni* and *C. coli* in stools. It is a very easy test to use and does not require any specific automation to be performed or to interpret the results, unlike molecular biology methods or some ELISAs. Furthermore, the performance results of CAMPYLOBACTER QUIK CHEK™ in this study are better than the ones reported in the literature for other immunochromatographic tests [11–13]*.* The main advantage of this CIDT is the rapidity in obtaining a result, enabling adapted medical care, if needed. Finally, this test should not replace culture which remains essential to perform antibiotic susceptibility testing, to assess isolates in order to obtain epidemiological information concerning outbreaks, and to detect *Campylobacter spp.* species that are not detected by the EIA. The place of this test in daily clinical practice needs to be evaluated.

## Data Availability

The datasets generated and/or analyzed during the current study are not publicly available due to their confidentiality, but are available from the corresponding author upon reasonable request.
